# We are talking about WM as a broad ability factor! Comment on Burgoyne, Frank, and Macnamara (2024)

**DOI:** 10.3758/s13423-025-02706-5

**Published:** 2025-05-13

**Authors:** Oliver Wilhelm, Jasmin Thelen, Florian Schmiedek

**Affiliations:** 1 Institute of Psychology and Education, Ulm University, Albert-Einstein-Allee 47, 89081 Ulm, Germany; 2 DIPF | Leibniz Institute for Research and Information in Education, Frankfurt, Germany

**Keywords:** Working memory, Reasoning, Reanalysis

## Abstract

Burgoyne, Frank, and Macnamara (*Psychonomic Bulletin & Review*, 2024) argued for a separation of updating and working memory factors. We agree that understanding variance across multiple different task classes and measures for the assessment of working memory is crucial. It is a strength of their contribution to include many and diverse subjects and also to study the convergent relation with fluid intelligence. In our view, however, their analysis and interpretation of findings is partly flawed, and other conclusions ought to be drawn from their data. More specifically, we argue that 1) the disengagement hypothesis is hardly convincing to account for their results, and 2) a reanalysis of the data supports other models more than the models put forward by Burgoyne et al. (2024).

Working memory (WM) is arguably best seen as a mean of the human mind to build, maintain, and change mental representations, which are in charge of ongoing thoughts and actions (Oberauer, 2020). Prior research has shown that factoring a broad variety of WM measures and relating them with similarly factored measures of fluid intelligence (gf) shows very strong relations—often near unity. A first set of prominent studies (Kyllonen & Christal, 1990) found the relation between WM and gf to range between 0.80 and 0.90. Improved analysis (Oberauer et al., 2005) of a popular meta-analysis (Ackerman et al., 2005) also found the relation between WM and gf to reach a magnitude of around 0.85. Many newer studies replicate this estimate (for instance, Monteiro et al., 2025).

Burgoyne, Frank, and Macnamara (BFM; 2024) suggest that different task classes of WM have differential validity for gf. In this commentary we argue that this suggestion is based on a weak theoretical argument and on a suboptimal data analysis. In this commentary we first consider a brief discussion of the disengagement hypothesis and then provide a reanalysis of the data from BFM.

The disengagement hypothesis according to BFM as an explanation for the covariance of *n*-back performance and gf posits that a common attentional mechanism underlying both is the removal of outdated information from WM. In the *n*-back task, this refers to no-longer-relevant elements, and in reasoning tasks, it refers to incorrect and therefore to-be-discarded hypotheses. Upon closer inspection, this assertion seems to be a substantial leap, and it lacks sufficient evidence to be considered a compelling explanation. While for WM updating tasks the speed of removing no-longer-relevant items from WM has been shown to be unrelated to overall WM performance (Ecker et al., 2014; Frischkorn et al., 2022), for reasoning tasks, removal of rejected hypotheses is not an essential part of performance at all (Johnson-Laird & Ragni, 2024). On the contrary, successful solution processes will typically require the modification of mental representations in accordance with mismatches that have been identified, while keeping track of which hypotheses already have been considered and flagged as incorrect. Insights gained from rejected hypotheses must be considered in subsequent deliberations. Compelling arguments for the disengagement hypothesis as an explanation for the shared variance between *n*-back and reasoning tasks would require evidence based on isolating and directly correlating individual differences in the removal processes on both sides—for example, using methods as proposed by Ecker et al. (2014).

BFM state in their abstract that they “found strong evidence for a dissociation between complex span and *n*-back tests, and more broadly, between working memory capacity and updating factors” (p.1). The latent variable model BFM eventually pursue distinguishes between the factors WM and Updating, with a reported correlation of latent factors of $\rho$= 0.57. Latent variable approaches aim at accounting for observed correlations by specifying parsimonious models. Amongst measures of cognitive ability, these observed correlations can be expected to show positive manifold—that is, be largely explained by a general factor. Over and above such a general factor, explanatory models can allow for relations specific to certain indicators. Inspection of the correlation matrix can point to instances of such specific relations. In the BFM correlation matrix amongst the Working Memory and Updating tasks, two correlations are particularly high. The spatial and letter *n*-back tasks are correlated at *r* = 0.71 and symmetry span and rotation span are correlated at *r* = 0.57. Other than that, the correlation matrix is generally positive and of approximately equal magnitude. At a quick glance, this correlation matrix did not at all suggest strong evidence for a dissociation between WM and updating and we thus reanalyzed the data from BFM.

We used the same data, estimators, missing data treatment, and cut-offs for fit indices as BFM (https://osf.io/q4pcw/?view_only=61e976d9d4a64b2ebef91cfa848c74ce). Our analysis was computed in R (Version 4.3.2; R Core Team, 2022), using *lavaan* (Version 0.6–17; Rosseel, 2012).

First, we specified a general factor model with one WM factor and two correlated errors for the two exceptionally high correlations mentioned above. Second, we added fluid intelligence to the WM model, just the way BFM did. Theoretically these models propose a common factor for all complex span, *n-*back, and other WM tasks used in this study. Additionally, the models allow for residual correlations between tasks that share certain specific task attributes. Symmetry span and rotation span are spatial complex span tasks that share the same overarching procedure of complex span tasks in the spatial domain. The two *n*-back tasks work the same way, except one uses letters and the other uses red squares in a grid. Moreover, they are composed of two kinds of tasks stacked onto each other. First, there is an updating requirement that is similar to the demands in the other two updating tasks (Keep Track and Tone Monitoring). Second, there is a speeded two-choice decision task requiring fast responses to each item. Such tasks demand setting response criteria for a speed–accuracy trade-off, which is likely to introduce individual differences in response caution into observed performance.

Table 1 provides fit of the models proposed by BFM and us (WTS). The WTS measurement model has one *df* less than the BFM model but much improved model fit. In the model predicting gf, the discrepancy between the BFM and the WTS model is larger and the fit improvement is substantial.

**Table 1:** Fit statistics for confirmatory factor analysis of the working memory task battery and its relation to fluid intelligence

Model		X^2^(*df*)	*RMSEA*	*CFI*	*AIC*	*BIC*
BFM-WM		248 (19)	0.097	0.919	24,204	24,333
WTS-WM		146 (18)	0.075	0.950	24,107	24,241
BFM-WM-gf		451 (51)	0.079	0.919	36,067	36,267
WTS-WM-gf		236 (51)	0.053	0.960	35,859	36,060

*N* = 1,272; BFM = model by Burgoyne, Frank, and Macnamara; WTS = model by Wilhelm, Thelen, and Schmiedek; WM = working memory; gf = fluid intelligence

Figure 1 presents the WTS-WM-gf model. Please note that the model presented here allows for correlated uniqueness between two pairs of variables. Equivalently, two nested factors for spatial complex span tasks and *n*-back tasks, respectively, could have been specified (and doing so provides no incremental explanation of gf).

**Fig. 1:**
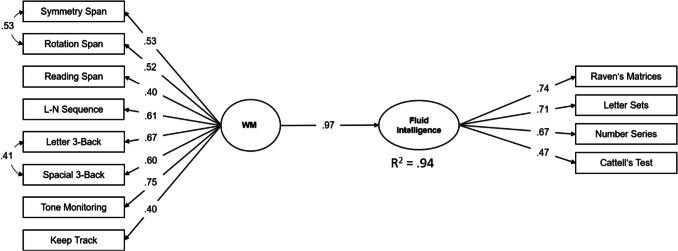
General factor model of working memory and its prediction of fluid intelligence (WTS-WM-gf)

Additional models are conceivable, and we provide some of these models in the OSF link of this paper (https://osf.io/vng6k/). Indeed, some of these models provide somewhat better fit to the data, but they might be tailored to the present data too much. There is also no gain in predictive validity over and above the model presented in Fig. 1. Taken together, the general factor WM model we propose is a) simple in explanatory terms, b) in line with earlier reports in the literature, c) highly predictive for key outcomes, and d) superior to a model that distinguishes two WM factors.

WM and gf are very highly correlated in the present data set, but the relation could not be constrained to unity without loss in fit. Despite the near perfect relation, we see a number of differences between WM and gf (Wilhelm & Kyllonen, 2021). First, WM tasks will always be somewhat speeded, given that all prominent WM tasks use time restrictions for stimulus exposition or response deadlines. Second, administration of WM tasks will always be computerized, whereas administration mode for gf varies, and arguably, within-administration-mode correlations are higher than between-administration-mode correlations. Third, knowledge is often helpful in gf tasks, but usually not in WM. For instance, vocabulary helps with verbal analogy tasks, and numerical facility helps with number series tasks. Fourth, gf tasks might stress aspects such as concept formation, which are irrelevant in WM tasks. There might be additional minor differences that jointly explain why WM and gf are not exactly, yet almost, the same.

Taken together, broad ability constructs such as WM or gf surpass most latent variables as they are prevalent in psychological journals in their generality, coverage, or nomological breadth. It is important to realize that WM transcends individual manifest variables or specific task classes in their meaning. It is similarly important that specific measures come with specific variance. Sometimes these specificities are located at the level of manifest variable and sometimes at the level of task classes (cf. Schmiedek et al., 2014). More often than not, methodological or procedural overlap of manifest variables is best considered swollen specific or nuisance variance (Cattell & Tsujioka, 1964; Reise et al., 2010; Spearman, 1927, 1929, 1939). BFM were substantiating such swollen specific variance in their two-factor model. Swollen specific variance is variance that can be attributed to peculiarities of two or more indicators that is of no substantive interest. Our model instead shows that a simpler model is in line with prior results from our and other research groups (Kyllonen & Christal, 1990; Schmiedek et al., 2009, 2014; Wilhelm et al., 2013).

## Data Availability

The dataset analyzed in this paper is available in the OSF repository of the original paper: https://osf.io/q4pcw/?view_only=61e976d9d4a64b2ebef91cfa848c74ce

## Code availability

The R code is available in the OSF repository: https://osf.io/vng6k/

## Declarations

We confirm that the work conforms to Standard 8 of the American Psychological Association’s Ethical Principles of Psychologists and Code of Conduct.

## References

[CR1] Ackerman, P. L., Beier, M. E., & Boyle, M. O. (2005). Working memory and intelligence: The same or different constructs? *Psychological Bulletin,**131*(1), 30–60. 10.1037/0033-2909.131.1.30 15631550 10.1037/0033-2909.131.1.30

[CR2] Burgoyne, A. P., Frank, D. J., & Macnamara, B. N. (2024). Which “working memory” are we talking about? Complex span tasks versus N-back. *Psychonomic Bulletin & Review*. 10.3758/s13423-024-02622-0 10.3758/s13423-024-02622-0 39633235

[CR3] Cattell, R. B., & Tsujioka, B. (1964). The importance of factor-trueness and validity, versus homogeneity and orthogonality, in test scales. *Educational and Psychological Measurement,**24*(1), 3–30. 10.1177/001316446402400101

[CR4] Ecker, U. K. H., Lewandowsky, S., & Oberauer, K. (2014). Removal of information from working memory: A specific updating process. *Journal of Memory and Language,**74*, 77–90. 10.1016/j.jml.2013.09.003

[CR5] Frischkorn, G. T., Von Bastian, C. C., Souza, A. S., & Oberauer, K. (2022). Individual differences in updating are not related to reasoning ability and working memory capacity. *Journal of Experimental Psychology: General,**151*(6), 1341–1357. 10.1037/xge0001141 35201837 10.1037/xge0001141

[CR6] Johnson-Laird, P. N., & Ragni, M. (2024). Reasoning about possibilities: Modal logics, possible worlds, and mental models. *Psychonomic Bulletin & Review*10.3758/s13423-024-02518-z 10.3758/s13423-024-02518-z PMC11836090 39012580

[CR7] Kyllonen, P. C., & Christal, R. E. (1990). Reasoning ability is (little more than) working-memory capacity?! *Intelligence,**14*(4), 389–433. 10.1016/S0160-2896(05)80012-1

[CR8] Monteiro, F., Nascimento, L. B., Leitão, J. A., Santos, E. J. R., Rodrigues, P., Santos, I. M., . . . Nascimento, C. S. (2025). Optimizing working memory assessment: Development of shortened versions of complex spans, updating, and binding tasks. *Psychological Research*, *89*(2), 65. 10.1007/s00426-025-02083-7 10.1007/s00426-025-02083-7 PMC11890332 40056259

[CR9] Oberauer, K. (2020). Towards a theory of working memory: From metaphors to mechanisms. In R. Logie, V. Camos, & N. Cowan (Eds.), *Working memory: State of the science* (pp. 116–149). Oxford University Press. 10.1093/oso/9780198842286.003.0005

[CR10] Oberauer, K., Schulze, R., Wilhelm, O., & Süß, H.-M. (2005). Working memory and intelligence—Their correlation and their relation: Comment on Ackerman, Beier, and Boyle (2005). *Psychological Bulletin,**131*(1), 61–65. 10.1037/0033-2909.131.1.61 15631551 10.1037/0033-2909.131.1.61

[CR11] R Core Team. (2022). *R: A language and environment for statistical computing* (Version 4.3.2) [Computer software]. https://www.R-project.org/

[CR12] Reise, S. P., Moore, T. M., & Haviland, M. G. (2010). Bifactor models and rotations: Exploring the extent to which multidimensional data yield univocal scale scores. *Journal of Personality Assessment,**92*(6), 544–559. 10.1080/00223891.2010.496477 20954056 10.1080/00223891.2010.496477 PMC2981404

[CR13] Rosseel, Y. (2012). lavaan: An R package for structural equation modeling. *Journal of Statistical Software*, *48*(2), 1–36. 10.18637/jss.v048.i02

[CR14] Schmiedek, F., Hildebrandt, A., Lövdén, M., Wilhelm, O., & Lindenberger, U. (2009). Complex span versus updating tasks of working memory: The gap is not that deep. *Journal of Experimental Psychology: Learning, Memory, and Cognition,**35*(4), 1089–1096. 10.1037/a0015730 19586272 10.1037/a0015730

[CR15] Schmiedek, F., Lövdén, M., & Lindenberger, U. (2014). A task is a task is a task: Putting complex span, n-back, and other working memory indicators in psychometric context. *Frontiers in Psychology*, *5*. 10.3389/fpsyg.2014.01475 10.3389/fpsyg.2014.01475 PMC4274887 25566149

[CR16] Spearman, C. (1927). Material versus abstract factors in correlation. *British Journal of Psychology. General Section,**17*(4), 322–326. 10.1111/j.2044-8295.1927.tb00434.x

[CR17] Spearman, C. (1929). The uniqueness of ‘G.’ *Journal of Educational Psychology,**20*(3), 212–216. 10.1037/h0072998

[CR18] Spearman, C. (1939). ‘Intelligence’ tests. *The Eugenics Review,**30*(4), 249–254. 21260327 PMC2985859

[CR19] Wilhelm, O., Hildebrandt, A., & Oberauer, K. (2013). What is working memory capacity, and how can we measure it? *Frontiers in Psychology*, *4*. 10.3389/fpsyg.2013.00433 10.3389/fpsyg.2013.00433 PMC3721021 23898309

[CR20] Wilhelm, O., & Kyllonen, P. (2021). To predict the future, consider the past: Revisiting Carroll (1993) as a guide to the future of intelligence research. *Intelligence,**89*, 101585. 10.1016/j.intell.2021.101585

